# Drug-Coated Balloon Angioplasty in Patients Undergoing Complex Percutaneous Coronary Intervention

**DOI:** 10.1016/j.jacasi.2024.04.007

**Published:** 2024-06-18

**Authors:** Hyun Sung Joh, Woochan Kwon, Doosup Shin, Seung Hun Lee, Young Joon Hong, David Hong, Sang Yoon Lee, Hanbit Park, Sunwon Kim, Sang Yeub Lee, Jin-Sin Koh, Hangyul Kim, Chan Joon Kim, Eun Ho Choo, Hyuck-Jun Yoon, Sang Don Park, Ki-Hyun Jeon, Jang-Whan Bae, Sung Gyun Ahn, Sung Eun Kim, Ki Hong Choi, Taek Kyu Park, Jeong Hoon Yang, Young Bin Song, Joo-Yong Hahn, Seung-Hyuk Choi, Hyeon-Cheol Gwon, Joo Myung Lee

**Affiliations:** a Department of Internal Medicine and Cardiovascular Center, Seoul National University Boramae Medical Center, Seoul National University College of Medicine, Seoul, Republic of Korea; b Samsung Medical Center, Sungkyunkwan University School of Medicine, Seoul, Republic of Korea; c Department of Cardiology, St. Francis Hospital and Heart Center, Roslyn, New York, USA; d Department of Internal Medicine and Cardiovascular Center, Chonnam National University Hospital, Chonnam National University Medical School, Gwangju, Republic of Korea; e Department of Cardiology, Gangneung Asan Hospital, University of Ulsan College of Medicine, Gangneung, Gangwon-do, Republic of Korea; f Department of Cardiology, Korea University Ansan Hospital, Ansan-si, Republic of Korea; g Chung-Ang University College of Medicine, Chung-Ang University Gwangmyeong Hospital, Gwangmyeong, Republic of Korea; h Gyeongsang National University School of Medicine, Gyeongsang National University Hospital, Jinju, Republic of Korea; i Uijeongbu St. Mary’s Hospital, The Catholic University of Korea, Seoul, Republic of Korea; j Seoul St. Mary’s Hospital, The Catholic University of Korea, Seoul, Korea; k Keimyung University Dongsan Hospital, Daegu, Republic of Korea; l Inha University Hospital, Incheon, Republic of Korea; m Division of Cardiovascular Medicine, Department of Internal Medicine, Seoul National University Bundang Hospital, Seongnam, Republic of Korea; n Department of Internal Medicine, College of Medicine, Chungbuk National University, Cheongju, Republic of Korea; o Yonsei University Wonju College of Medicine, Wonju Severance Christian Hospital, Wonju, Republic of Korea; p Department of Medicine, Inje University Ilsan Paik Hospital, Goyang, Republic of Korea

**Keywords:** de novo, drug-coated balloon, drug-eluting stent(s), complex percutaneous coronary intervention

## Abstract

**Background:**

There are limited clinical data on drug-coated balloon (DCB)-based percutaneous coronary intervention (PCI) compared with drug-eluting stent (DES)-only PCI in patients with complex coronary artery lesions.

**Objectives:**

The goal of the current study was to investigate the efficacy of DCB in patients undergoing PCI for complex coronary artery lesions.

**Methods:**

From an institutional registry of patients with de novo complex coronary artery lesions, 126 patients treated with DCB-based PCI were compared with 234 propensity score–matched patients treated with DES-only PCI. Complex coronary artery lesions were defined as the presence of at least 1 of the following: bifurcation, chronic total occlusion, unprotected left main disease, long lesion ≥38 mm, multivessel disease, lesion requiring ≥3 devices, or severe calcification. The primary endpoint was target vessel failure (TVF) at 2 years, a composite of cardiac death, target vessel–related myocardial infarction, and target vessel revascularization.

**Results:**

Baseline characteristics were comparable between the 2 groups. DCB-based PCI showed a comparable risk of TVF vs DES-based PCI (7.6% vs 8.1%; HR: 0.81; 95% CI: 0.33-1.99; *P* = 0.638). The risks of cardiac death (5.0% vs 5.7%; HR: 0.78; 95% CI: 0.24-2.49), target vessel–related myocardial infarction (0.9% vs 1.3%; HR: 2.65; 95% CI: 0.26-27.06), and target vessel revascularization (3.5% vs 2.0%; HR: 1.30; 95% CI: 0.30-5.67) were also comparable between the 2 groups.

**Conclusions:**

DCB-based PCI showed comparable risks of TVF vs those of DES-only PCI in patients with complex coronary artery lesions. DCB might be considered as a suitable alternative device to DES in patients undergoing complex PCI. (Long-term Outcomes and Prognostic Factors in Patient Undergoing CABG or PCI; NCT03870815)

Percutaneous coronary intervention (PCI) of complex coronary artery lesions accounts for >30% of all contemporary PCI procedures.^1^ Despite the use of new-generation drug-eluting stents (DES), the risk of adverse clinical events is still higher in patients undergoing complex PCI than in those undergoing noncomplex PCI.^2^^,^^3^ In addition to a higher burden of cardiovascular risk factors and residual coronary atherosclerosis, procedural factors, including longer and multiple stents, are associated with an increased risk of adverse clinical events due to a higher risk of suboptimal stent implantation or delayed endothelization after complex PCI compared with noncomplex PCI.^4^

Drug-coated balloons (DCBs) were developed under the concept of angioplasty without implantation of metal and designed as a semi-compliant balloon coated with antiproliferative agents that are delivered to the target vessel wall during inflation.^5^ Considering that DES implantation is inherently associated with a 2% annual risk of late stent-related events, use of DCB has been considered as an alternative strategy that could reduce the extent and number of DES used during PCI. Nevertheless, clinical evidence of DCB use in the treatment of coronary artery disease (CAD) has mostly been confined to in-stent restenosis (ISR). Recently, a few observational studies compared clinical outcomes between DCB- and DES-based revascularization strategies in the treatment of specific subsets of complex coronary artery lesions such as bifurcation, diffuse disease, and multivessel CAD.^6^, ^7^, ^8^ However, the results are conflicting, and evidence regarding DCB use in various complex coronary artery lesions remains limited.

The goal of the current study was to investigate the efficacy of DCB-based PCI in patients undergoing PCI for various complex coronary artery lesions.

## Methods

This was a retrospective analysis of a prospective institutional registry from a tertiary referral center in the Republic of Korea. Patients were selected from 2 institutional registries of Samsung Medical Center (Seoul, Republic of Korea), a registry of PCI in which DCBs have been used, enrolling from January 2016 to December 2019, and another registry of DES use, enrolling from January 2012 to December 2015. Previous reports were published using part of the DES registry.^9^^,^^10^ A total of 1,940 patients who had a complex coronary artery lesion and underwent PCI were selected.

**Figure 1:**
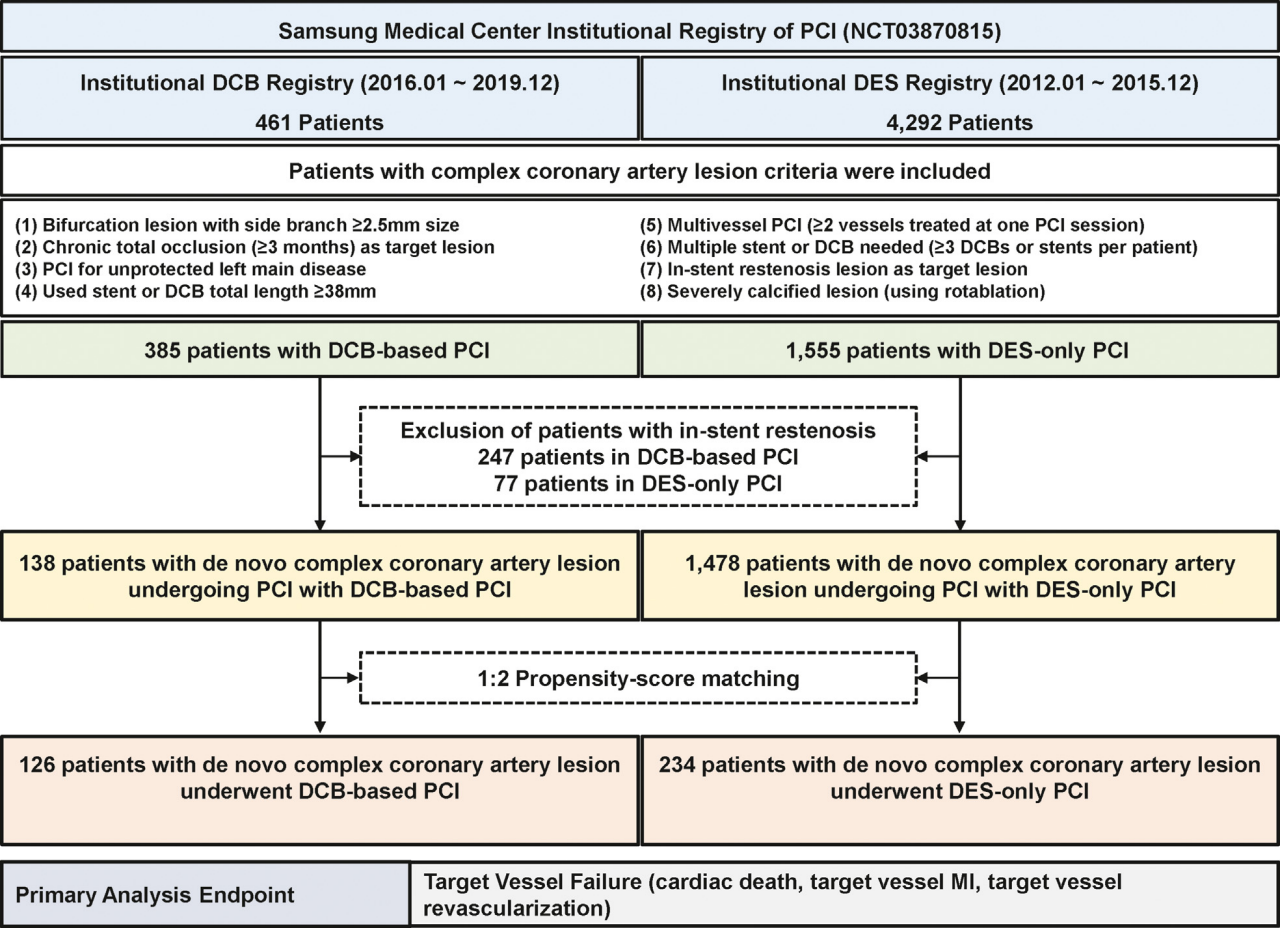
Study Flow Study flow is presented. A total of 1,940 patients who had a complex coronary artery lesion were included from a prospective institutional registry. Among them, 324 patients with in-stent restenosis were excluded, leaving 138 patients with drug-coated balloon (DCB)-based percutaneous coronary intervention (PCI) and 1,478 patients with drug-eluting stent (DES)-only PCI. Propensity score matching with a 1:2 ratio was used for adjustment of baseline characteristics differences, leaving 126 patients with DCB-based PCI and 234 patients with DES-only PCI for analysis. MI = myocardial infarction.

Complex coronary artery lesions were defined as 1 of the following: 1) bifurcation lesions with side branch diameter ≥2.5 mm; 2) chronic total occlusions (CTOs) with an occlusion duration ≥3 months; 3) unprotected left main (LM) disease; 4) long lesions (used stent or DCB length ≥38 mm; 5) multivessel PCI (≥2 major epicardial coronary vessels treated at 1 PCI session); 6) multiple devices used (≥3 DCBs or stents per patient); 7) ISR lesion; or 8) severely calcified lesion (requiring a rotablation). For the purposes of the current study, 324 patients with ISR were excluded. Patients whose PCI involved use of DCB (thus, both DCB-only and DCB and DES hybrid PCI, including bail-out DES implantation due to suboptimal DCB angioplasty) were classified into the DCB-based PCI group (n = 138) and the others into the DES-only PCI group (n = 1,478). To adjust for the significant differences of baseline characteristics between the 2 groups, 126 patients with DCB-based PCI were matched with 234 patients with DES-only PCI using propensity scores (Figure 1).

The study protocol was approved and the requirement for informed consent was waived by the Institutional Review Board of Samsung Medical Center. The current study was conducted according to the principles of the Declaration of Helsinki. The institutional cardiovascular catheterization database of Samsung Medical Center is registered at ClinicalTrials.gov (NCT03870815).

### Procedures of PCI

All interventions were performed by using standard techniques. A loading dose of aspirin 300 mg was given before PCI, unless patients were already receiving aspirin therapy for at least 7 days. A loading dose of clopidogrel 300 mg was given 24 hours before PCI or 600 mg was given on the day of PCI, unless patients were already taking clopidogrel for at least 5 days. A loading dose of prasugrel 60 mg or ticagrelor 180 mg was given before PCI in selected patients with acute coronary syndrome (ACS), unless patients were already receiving potent P2Y_12_ inhibitor therapy for at least 7 days. Unfractionated heparin or low-molecular-weight heparin was used for procedural anticoagulation according to standard protocol.

In the DCB-based PCI group, all DCBs used were paclitaxel-coated balloons, and procedures were performed according to international DCB consensus.^11^^,^^12^ The target lesion was routinely predilated by using an optimal-sized balloon with a balloon-to-reference vessel ratio of 0.8 to 1.0. Use of specialty balloons, including scoring, cutting, or noncompliant balloons, was allowed per operator discretion during pre-dilatation. If there was a flow-limiting dissection or >30% residual stenosis after lesion preparation, bail-out stent implantation was recommended rather than using DCB. After appropriate lesion preparation, DCB was inflated for at least 60 seconds with its nominal pressure. In cases with flow-limiting dissections or significant residual stenosis after DCB PCI, additional bail-out stenting was allowed.

In the DES-only PCI group, all implanted DES were new-generation DES, and procedures were performed with the use of current standard techniques. Type of DES, stenting techniques, use of intravascular imaging devices, and need for DES postdilatation were left to operator discretion. After the index procedure, it was recommended that all patients take aspirin and a P2Y_12_ inhibitor; the duration of dual antiplatelet therapy was left up to physician discretion, however.

### Data Collection, Follow-Up, and Study Endpoints

Baseline characteristics, angiographic and procedural data, and clinical outcomes were collected prospectively in the institutional registry. Additional information was obtained from medical records and telephone contacts, if necessary. Coronary angiograms were reviewed, and quantitative coronary angiography was performed by an independent core laboratory at Samsung Medical Center.

The primary analysis endpoint was target vessel failure (TVF), a composite of cardiac death, target vessel–related myocardial infarction (MI), and ischemia-driven target vessel revascularization at 2 years of follow-up. The secondary analysis endpoint included all-cause death, any MI (including nontarget vessel territory), any revascularization, target lesion revascularization (TLR), definite or probable device thrombosis, and stroke (ischemic or hemorrhagic). Procedure-related MI was not included as a clinical event in the current analysis. Spontaneous MI was defined according to the third universal definition for MI.^13^ Death of unknown cause was assumed to be cardiac related according to the definitions of the Academic Research Consortiums.^14^ Both target vessel revascularization and target lesion revascularization were clinically driven. They were defined as revascularization at the previously treated segment from 5 mm proximal to the stent to 5 mm distal to the stent with ≥70% diameter stenosis and at least 1 of the following: 1) recurrence of angina; 2) positive noninvasive test result; or 3) positive invasive physiological test result. Definite, probable, and possible stent thromboses were defined according to the definitions of the Academic Research Consortiums. The mortality data for patients lost to follow-up were confirmed by National Death Records. All clinical events were adjudicated by expert interventional cardiologists blinded to treatment strategy.

### Statistical Analysis

All data were analyzed on a per-patient basis. Vessels with the most severe stenosis were selected as the representative vessel in patients with multivessel PCI at the time of the index procedure. Additional lesion-level analysis of target lesion characteristics was performed to compare lesion complexity between the 2 groups. All discrete and categorical variables are presented as numbers and relative frequencies (percentages). Continuous variables are expressed as mean ± SD or median (IQR) according to their distribution, checked by using the Kolmogorov-Smirnov test and visual inspection of a Q-Q plot. Categorical variables were compared by using the chi-square test, and continuous variables were compared by using Student’s *t*-test or Mann-Whitney *U* test according to their distribution.

To adjust for uneven distribution of baseline characteristics according to treatment strategies, 1:2 propensity score matching was performed using a caliper width of 0.1 between the 2 groups. Propensity score was calculated by using logistic regression, with variables including age, sex, hypertension, diabetes mellitus, hyperlipidemia, current smoker, chronic kidney disease, previous MI, previous PCI, previous coronary artery bypass graft, ACS, extent of disease, and multivessel disease. The covariate balance after propensity score matching was measured by calculating the absolute standard mean differences. Absolute standard mean differences were within 10% across all matched covariables, suggesting balance achievement between the 2 groups (Supplemental Table 1). In addition, inverse probability–weighted, propensity score–adjusted, and propensity score–stratified Cox proportional hazards regression analyses were conducted for sensitivity analysis in the original population.

The cumulative incidence of clinical events is presented as Kaplan-Meier estimates and were compared by using a log-rank test. Stratified multivariable Cox proportional hazards regression with matched pairs as strata was used to calculate adjusted HRs and 95% CIs to compare the risk of clinical events between the matched groups. The assumption of proportionality was assessed by using Schoenfeld residuals and graphically by the log-log plot. The adjusted covariables were age, sex, diabetes mellitus, clinical presentation, target vessel location, multivessel PCI, PCI of CTO lesion, PCI of unprotected LM disease, and concomitant use of aspirin and a P2Y_12_ inhibitor. Subgroup analysis of the primary outcome was performed according to clinical and lesion characteristics of interest between the 2 groups. The interaction between treatment effect and the covariables was evaluated by a Cox proportional hazards regression model. A multivariable Cox proportional hazards model was used to identify independent predictors for TVF in the unmatched population. All probability values were two-sided, and *P* values <0.05 were considered statistically significant.

Statistical analyses were performed by using SPSS 20.0 for Windows (IBM SPSS Statistics, IBM Corporation) and R version 4.3.0 (R Foundation for Statistical Computing).

## Results

### Baseline Characteristics

Table 1 presents baseline characteristics of the original and propensity score–matched populations. Before matching, patients who underwent DCB-based PCI were older, were current smokers, and had a higher proportion of dyslipidemia, previous MI, previous PCI, 3-vessel disease, and multivessel disease. After matching, demographic characteristics, cardiovascular risk factors, and angiographic disease severity were similar between the 2 groups (Supplemental Table 1). Among the total population, 100 patients (27.8%) presented with ACS, 294 patients (81.7%) had multivessel disease, and 50 patients (13.9%) had unprotected LM disease.

**Table 1:** Comparison of Baseline Characteristics According to Treatment Strategy

	Original Population				Matched Population			
	Total	DCB-Based PCI	DES-Only PCI	*P* Value	Total	DCB-Based PCI	DES-Only PCI	*P* Value
No. of patients	1,616	138 (8.5)	1,478 (91.5)		360	126 (35.0)	234 (65.0)	
Demographic characteristics								
Age, y	64.2 ± 11.2	66.8 ± 10.9	63.7 ± 11.2	0.004	66.4 ± 10.8	66.2 ± 10.8	66.6 ± 10.8	0.719
Male	1,267 (78.4)	112 (81.2)	1,155 (78.1)	0.475	294 (81.7)	102 (81.0)	192 (82.1)	0.909
Body mass index, kg/m^2^	24.6 ± 3.1	24.5 ± 3.1	24.6 ± 3.1	0.834	24.5 ± 3.0	24.5 ± 3.1	24.5 ± 3.0	0.908
Baseline LVEF, %	59.5 ± 10.0	58.7 ± 12.2	59.6 ± 9.8	0.413	59.2 ± 11.2	58.9 ± 12.1	59.3 ± 10.8	0.392
Cardiovascular risk factor								
Hypertension	999 (61.8)	96 (69.6)	903 (61.1)	0.062	254 (70.6)	86 (68.3)	168 (71.8)	0.561
Diabetes mellitus	885 (54.8)	74 (53.6)	811 (54.9)	0.847	199 (55.3)	68 (54.0)	131 (56.0)	0.798
Hyperlipidemia	588 (36.4)	96 (69.6)	492 (33.3)	<0.001	232 (64.4)	84 (66.7)	148 (63.2)	0.595
Current smoker	823 (50.9)	87 (63.0)	736 (49.8)	0.004	215 (59.7)	78 (61.9)	137 (58.5)	0.612
Chronic kidney disease	108 (6.7)	11 (8.0)	97 (6.6)	0.649	34 (9.4)	9 (7.1)	25 (10.7)	0.364
Peripheral artery disease	53 (3.3)	5 (3.6)	48 (3.2)	>0.999	13 (3.6)	5 (4.0)	8 (3.4)	>0.999
Previous myocardial infarction	136 (8.4)	25 (18.1)	111 (7.5)	<0.001	47 (13.1)	17 (13.5)	30 (12.8)	0.987
Previous PCI	232 (14.4)	51 (37.0)	181 (12.2)	<0.001	101 (28.1)	39 (31.0)	62 (26.5)	0.438
Previous CABG	47 (2.9)	2 (1.4)	45 (3.0)	0.432	7 (1.9)	2 (1.6)	5 (2.1)	>0.999
Family history of CAD	184 (11.4)	20 (14.5)	164 (11.1)	0.289	49 (13.6)	18 (14.3)	31 (13.2)	0.910
Clinical presentation				0.154				0.388
Acute coronary syndrome	600 (37.1)	43 (31.2)	557 (37.7)		100 (27.8)	39 (31.0)	61 (26.1)	
Stable ischemic heart disease	1,016 (63.0)	95 (68.8)	921 (62.3)		260 (72.2)	87 (69.0)	173 (73.9)	
Discharge medication								
Aspirin	1,495 (92.5)	135 (97.8)	1,360 (92.0)	0.021	337 (93.6)	124 (98.4)	213 (91.0)	0.012
P2Y_12_ inhibitor	1514 (93.7)	135 (97.8)	1,379 (93.3)	0.056	314 (94.7)	123 (97.6)	218 (93.2)	0.120
Beta-blocker	697 (43.1)	48 (34.8)	649 (43.9)	0.048	140 (38.9)	44 (34.9)	96 (41.0)	0.308
RAAS blockade	746 (46.2)	61 (44.2)	685 (46.3)	0.694	168 (46.7)	55 (43.7)	113 (48.3)	0.465
Statin	1,487 (92.0)	126 (91.3)	1,361 (92.1)	0.874	332 (92.2)	116 (92.1)	216 (92.3)	>0.999
Angiographic evaluation								
Extent of disease				0.035				0.933
1-vessel disease	397 (24.6)	23 (16.7)	374 (25.3)		66 (18.3)	23 (18.3)	43 (18.4)	
2-vessel disease	722 (44.7)	62 (44.9)	660 (44.7)		167 (46.4)	57 (45.2)	110 (47.0)	
3-vessel disease	497 (30.8)	53 (38.4)	444 (30.0)		127 (35.3)	46 (36.5)	81 (34.6)	
Multivessel disease	1,219 (75.4)	115 (83.3)	1,104 (74.7)	0.031	294 (81.7)	103 (81.7)	191 (81.6)	>0.999
LM disease	217 (13.4)	19 (13.8)	198 (13.4)	>0.999	50 (13.9)	17 (13.5)	33 (14.1)	>0.999

Values are mean ± SD or n (%) unless otherwise indicated.

CABG = coronary artery bypass graft; CAD = coronary artery disease; DCB = drug-coated balloon; DES = drug-eluting stent(s); LM = left main; LVEF = left ventricular ejection fraction; PCI = percutaneous coronary intervention; RAAS = renin-angiotensin-aldosterone system.

### Target Vessel, Target Lesion, and Procedural Characteristics

Table 2 presents target vessel and lesion characteristics in the matched population. Among the total target lesions (n = 630), 70.6% were type B2/C lesions, 2.1% were culprit lesions of ACS, 20.1% were bifurcation lesions, 45.6% were diffuse, 2.2% were thrombotic, 13.2% were CTO, 12.0% were ostial lesions, and 12.6% had significant calcification. Patients with DCB-based PCI had higher proportions of CTO and ostial lesions than those with DES-only PCI. Conversely, the DCB-based PCI group had a lower proportion of unprotected LM disease than the DES-only PCI group. Although baseline stenosis severity and the total length of target lesions were comparable between the 2 groups, post-PCI diameter stenosis was higher in the DCB-based PCI group than in the DES-only PCI group.

**Table 2:** Comparison of Target Vessel and Lesion Characteristics According to Treatment Strategy in Matched Population

	Total (N = 360)	DCB-Based PCI (n = 126)	DES-Only PCI (n = 234)	*P* Value
Target vessel location				<0.001
LAD	137 (38.1)	41 (32.5)	96 (41.0)	
LCX	95 (26.4)	57 (45.2)	38 (16.2)	
RCA	96 (26.7)	26 (20.6)	70 (29.9)	
LM	32 (8.9)	2 (1.6)	30 (12.8)	
No. of target lesion(s) (per patient)	1.8 ± 0.9	1.5 ± 0.7	1.9 ± 1.0	<0.001
Total no. of target lesions	630	185	445	
Target lesion characteristics (per lesion)				
ACC/AHA type B2/C	441 (70.6)	138 (74.6)	303 (68.9)	0.181
Culprit lesion of ACS	13 (2.1)	2 (1.1)	11 (2.5)	0.412
Bifurcation lesion	126 (20.1)	36 (19.5)	90 (20.4)	0.882
Non-LM true bifurcation lesion	49 (7.8)	14 (7.6)	35 (7.9)	>0.999
Diffuse lesion (lesion length ≥20 mm)	284 (45.6)	95 (51.4)	189 (43.2)	0.073
Thrombotic lesion	14 (2.2)	3 (1.6)	11 (2.5)	0.709
Chronic total occlusion	83 (13.2)	38 (20.5)	45 (10.1)	0.001
Ostial lesion	75 (12.0)	30 (16.2)	45 (10.2)	0.047
Calcification (moderate or severe)	79 (12.6)	16 (8.6)	63 (14.2)	0.074
Pre-PCI mean diameter stenosis, %	86.0 ± 10.4	87.3 ± 11.0	85.2 ± 9.9	0.049
Post-PCI mean diameter stenosis, %	4.6 ± 9.1	6.2 ± 9.5	3.7 ± 8.8	<0.001
Mean lesion length of target vessel, mm	23.6 ± 13.4	24.2 ± 13.0	23.2 ± 13.6	0.156
Total lesion length of target vessel, mm	39.7 ± 28.2	36.7 ± 27.2	41.3 ± 28.7	0.052

Values are n (%) or mean ± SD unless otherwise indicated.

ACC = American College of Cardiology; ACS = acute coronary syndrome; AHA = American Heart Association; LAD = left anterior descending; LCX = left circumflex; RCA = right coronary artery; other abbreviations as in Table 1.

**Figure 2:**
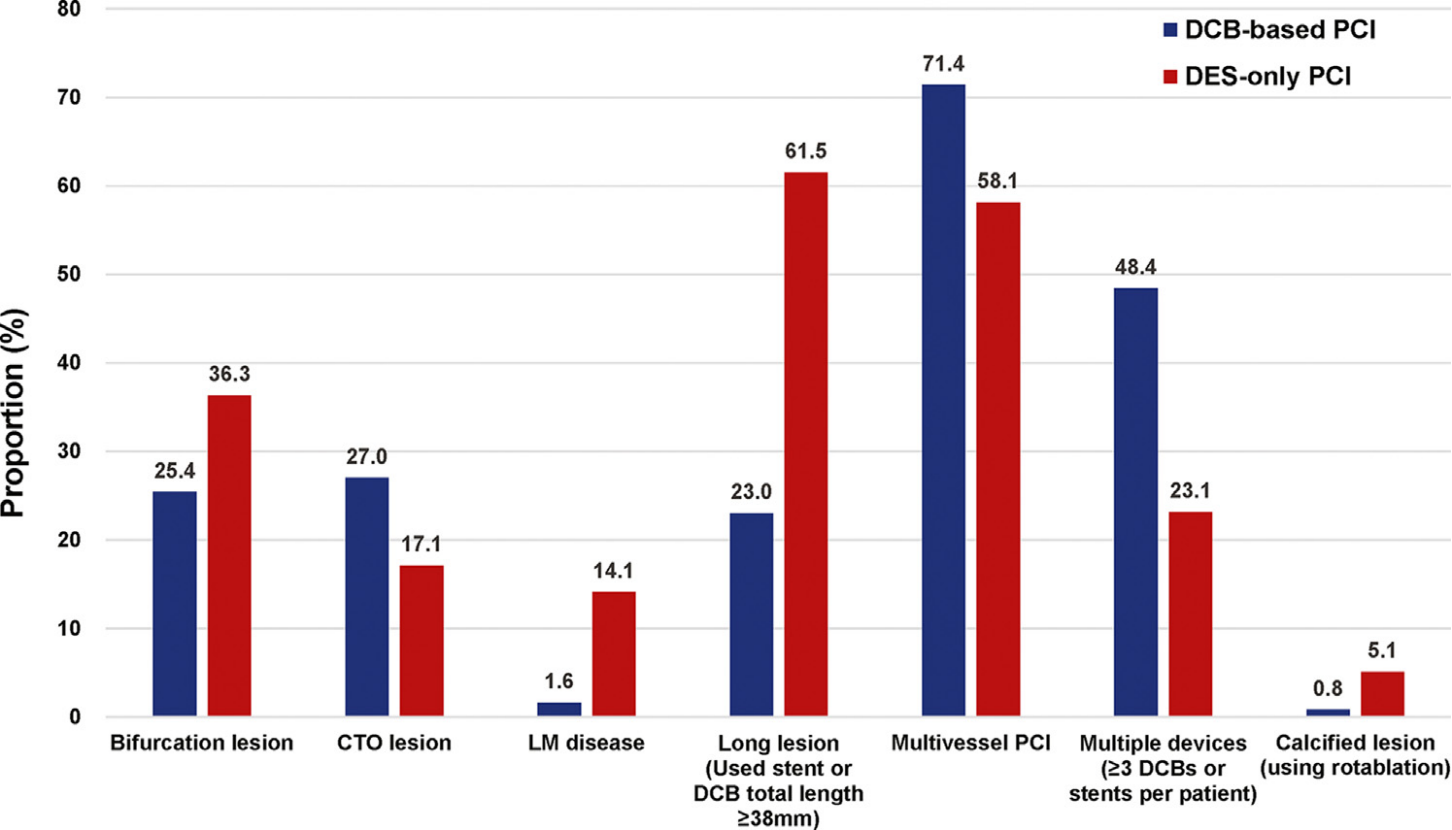
Proportion of Complex Coronary Artery Lesions in the Study Population Bar graphs show the proportion of complex coronary artery lesions between the DCB-based and DES-only PCI groups. Blue bars denote the proportion of DCB-based PCI, and the red bars denote the proportion of DES-only PCI. CTO = chronic total occlusion; LM = left main; other abbreviations as in Figure 1.

Table 3 summarizes procedural characteristics in the matched population. Compared with the DES-only PCI group, DCB-based PCI was more frequently performed in multivessel CAD and CTO. Conversely, unprotected LM disease was treated more with DES-only PCI (Figure 2). The mean number of devices used (2.6 ± 1.3 vs 1.9 ± 0.9) was higher, but mean diameter (2.62 ± 0.32 mm vs 3.00 ± 0.42 mm) and total length of devices used (43.8 ± 29.0 mm vs 48.8 ± 26.1 mm) were lower in the DCB-based group than in the DES-only group. Comparisons of target vessel and procedural characteristics in the original population are provided in Supplemental Tables 2 and 3.

**Table 3:** Comparison of Procedural Characteristics According to Treatment Strategy in the Matched Population

	Total (N = 360)	DCB-Based PCI (n = 126)	DES-Only PCI (n = 234)	*P* Value
Total fluoroscopy time, min	23.5 ± 14.5	26.1 ± 15.9	21.4 ± 13.4	<0.001
Multivessel PCI	233 (64.7)	92 (73.0)	141 (60.3)	0.021
Use of intravascular imaging	124 (34.4)	68 (54.0)	56 (23.9)	<0.001
PCI of CTO lesion	74 (20.6)	34 (27.0)	40 (17.1)	0.038
PCI of bifurcation lesion	121 (33.6)	35 (27.8)	86 (36.8)	0.109
PCI of unprotected LM disease	38 (10.6)	2 (1.6)	36 (15.4)	<0.001
PCI of thrombotic lesion	13 (3.6)	2 (1.6)	11 (4.7)	0.225
Use of rotational atherectomy	11 (3.1)	1 (0.8)	10 (4.3)	0.131
Mean number of devices used	2.1 ± 1.1	2.6 ± 1.3	1.9 ± 0.9	<0.001
DCB	0.4 ± 0.6	1.2 ± 0.5	0.0 ± 0.0	<0.001
DES	1.7 ± 1.1	1.5 ± 1.3	1.9 ± 0.9	0.003
Adjunctive dilatation	106 (29.4)	22 (17.5)	84 (35.9)	<0.001
Mean diameter of devices used in target lesion, mm	2.86 ± 0.42	2.62 ± 0.32	3.00 ± 0.42	<0.001
Total length of devices used in target lesion, mm	47.0 ± 27.2	43.8 ± 29.0	48.8 ± 26.1	0.009
Post-PCI TIMI flow grade 3	350 (97.2)	124 (98.4)	226 (96.6)	0.501
Bailout stenting	4 (1.1)	4 (3.2)	0 (0.0)	0.027
Procedure-related complications	12 (3.3)	4 (3.2)	8 (3.4)	>0.999
No reflow or slow flow	6 (1.7)	4 (3.2)	2 (0.9)	0.227
Cardiogenic shock	1 (0.3)	0 (0.0)	1 (0.4)	>0.999
Cardiopulmonary resuscitation	0 (0.0)	0 (0.0)	0 (0.0)	>0.999
Defibrillation	0 (0.0)	0 (0.0)	0 (0.0)	>0.999
Use of IABP	3 (0.8)	0 (0.0)	3 (1.3)	0.504
Use of PCPS	2 (0.6)	0 (0.0)	2 (0.9)	0.766
Stroke	1 (0.3)	0 (0.0)	1 (0.4)	>0.999
Procedural success	354 (98.3)	122 (96.8)	232 (99.1)	0.227

Values are mean ± SD or n (%).

CTO = chronic total occlusion; IABP = intra-aortic balloon pump; PCPS = percutaneous cardiopulmonary support; other abbreviations as in Table 1.

### Clinical Outcomes According to Treatment Strategy

**Figure 3:**
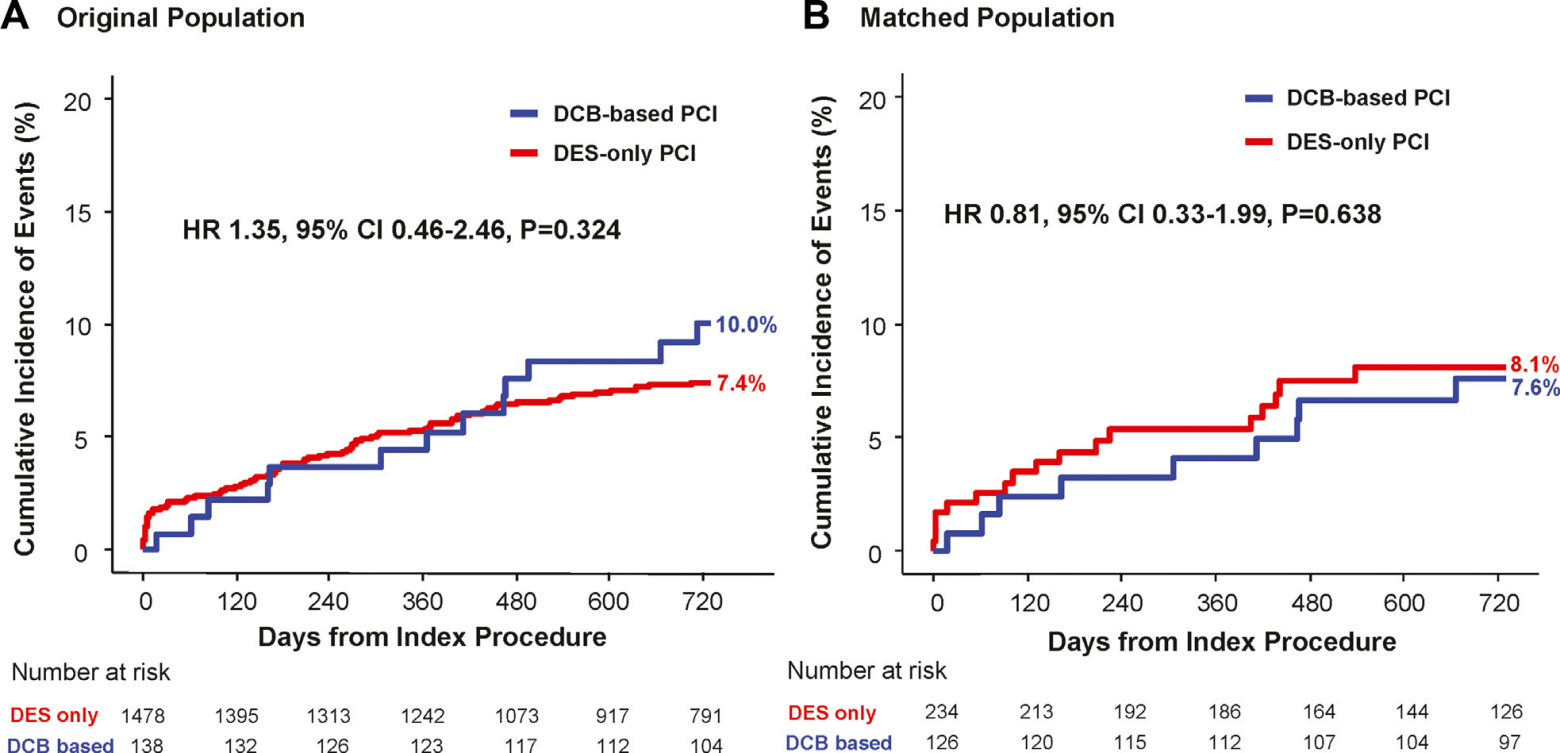
Comparison of Target Vessel Failure Between DCB-Based vs DES-Based PCI Kaplan-Meier curve is presented for cumulative incidence of target vessel failure at 2 years between the DCB-based PCI group and the DES-only PCI group in the original population (A) and in the matched population (B). Stratified multivariable Cox proportional hazards regression was used to calculate adjusted HRs and 95% CIs. The adjusted covariables were age, sex, diabetes mellitus, clinical presentation, target vessel location, multivessel PCI, PCI of CTO lesion, PCI of unprotected LM disease, and concomitant use of aspirin and P2Y_12_ inhibitor. Abbreviations as in Figures 1 and 2.

Table 4 presents a comparison of clinical outcomes between the DCB-based and DES-only PCI groups in the matched population. The cumulative incidence of TVF at 2 years was comparable between the 2 groups in both the original population (DCB-based 10.0% vs DES-only 7.4%; HR: 1.35; 95% CI: 0.74-2.46; *P* = 0.324) and the matched population (7.6% vs 8.1%; HR: 0.81; 95% CI: 0.33-1.99; *P* = 0.638) (Figure 3). Similarly, the risk of cardiac death (5.0% vs 5.7%; HR: 0.78; 95% CI: 0.24-2.49; *P* = 0.668), target vessel–related MI (0.9% vs 1.3%; HR: 2.65; 95% CI: 0.26-27.06; *P* = 0.412), and target vessel revascularization (3.5% vs 2.0%; HR: 1.30; 95% CI: 0.30-5.67; *P* = 0.732) were comparable between the 2 groups in the matched population. Device thrombosis occurred only in the DES-only PCI group.

**Table 4:** Comparison of Clinical Outcomes at 2 Years According to Treatment Strategy in Matched Population

	DCB-Based PCI	DES-Only PCI	Univariable HR (95% CI)	*P* Value	Multivariable HR^a^ (95% CI)	*P* Value
Target vessel failure	9 (7.6%)	17 (8.1%)	0.97 (0.43-2.22)	0.791	0.81 (0.33-1.99)	0.638
All-cause death	13 (10.8%)	18 (8.8%)	1.22 (0.58-2.57)	0.633	1.06 (0.46-2.41)	0.898
Cardiac death	6 (5.0%)	12 (5.7%)	0.88 (0.33-2.37)	0.749	0.78 (0.24-2.49)	0.668
Any myocardial infarction	2 (1.7%)	3 (1.3%)	1.33 (0.22-7.98)	0.852	5.33 (0.84-33.62)	0.075
Target vessel myocardial infarction	1 (0.9%)	3 (1.3%)	0.67 (0.07-6.41)	0.647	2.65 (0.26-27.06)	0.412
Any vessel revascularization	7 (6.0%)	6 (3.2%)	2.25 (0.70-7.18)	0.229	1.43 (0.46-4.49)	0.539
Target vessel revascularization	4 (3.5%)	4 (2.0%)	2.21 (0.49-10.0)	0.483	1.30 (0.30-5.67)	0.732
Target lesion revascularization	3 (2.6%)	2 (1.0%)	5.16 (0.53-50.4)	0.307	3.07 (0.35-26.80)	0.309
Definite or probable device thrombosis	0 (0.0%)	5 (2.2%)	NA	NA	NA	NA
Stroke (ischemic or hemorrhagic)	4 (3.3%)	4 (1.9%)	1.69 (0.42-6.85)	0.425	1.34 (0.27-6.56)	0.716

NA = not applicable; other abbreviations as in Tables 1 and 3.

a Adjusted covariables were age, male, diabetes mellitus, clinical presentation, target vessel location, multi-vessel PCI, PCI of CTO lesion, PCI of unprotected LM disease, and concomitant use of aspirin and P2Y_12_ inhibitor.

**Figure 4:**
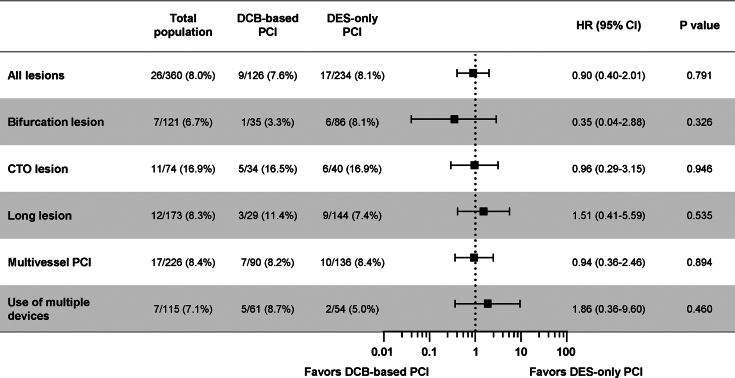
Comparison of Target Vessel Failure According to Lesion Characteristics Cumulative incidence and HR with 95% CI of target vessel failure at 2 years are presented between DCB-based and DES-only PCI groups according to various lesion characteristics. The HRs were calculated with the DES-only group as a reference. The risk of target vessel failure at 2 years was comparable between the 2 groups across various complex coronary artery lesions without significant interactions. Abbreviations as in Figures 1 and 2.

The 2-year risk of TVF was comparable between the 2 groups across various complex coronary artery lesions (Figure 4). The treatment effect of DCB-based PCI was also comparable with DES-only PCI across various clinical characteristics without significant interaction (Supplemental Figure 1). In a multivariable analysis of the original population, the independent predictors for TVF were diabetes mellitus, chronic kidney disease, and LM disease but not DCB-based PCI (HR: 1.18, 95% CI: 0.59-2.36; *P* = 0.643) (Supplemental Table 4). Sensitivity analyses using inverse probability–weighted and propensity scores also showed comparable risk of TVF between the 2 groups (Supplemental Table 5).

## Discussion

In the current propensity score–matched cohort study, DCB-based PCI showed comparable risk of TVF at 2 years with DES-only PCI in patients with de novo complex coronary artery lesions. The comparable risk of TVF was consistently observed in various subgroups of complex coronary artery lesions and clinical characteristics (Central Illustration).

**Central Illustration:**
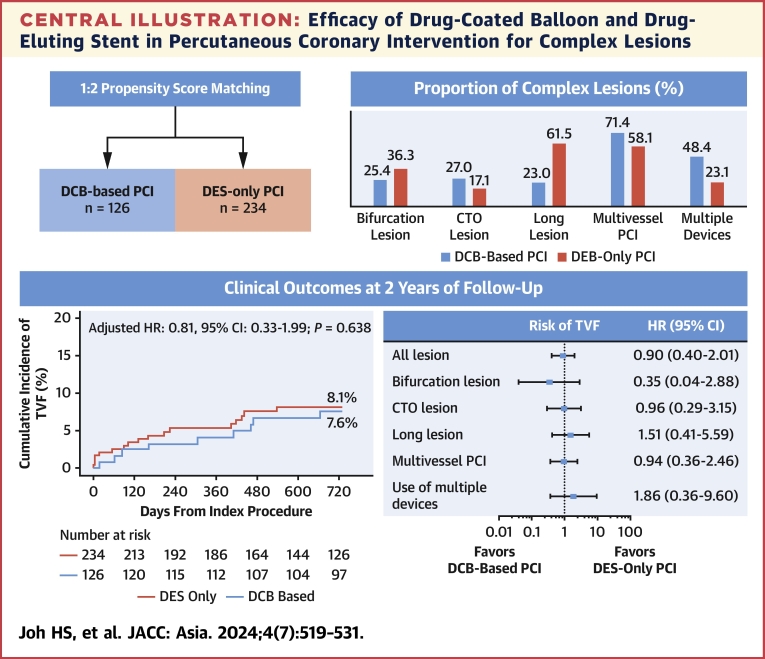
Efficacy of Drug-Coated Balloon and Drug-Eluting Stent in Percutaneous Coronary Intervention for Complex Lesions The current study evaluated clinical outcomes of drug-coated balloon (DCB)-based percutaneous coronary intervention (PCI) compared with drug-eluting stent (DES)-only PCI in patients undergoing PCI for various complex coronary lesions. DCBs were more frequently used for chronic total occlusion (CTO), multivessel coronary artery disease, and lesions requiring multiple devices. DCB-based PCI showed comparable risk of target vessel failure (TVF) at 2 years with DES-only PCI in patients with de novo complex coronary artery lesions. The comparable risk of TVF was consistently observed in various subgroups of complex coronary artery lesions.

DCB-based PCI without leaving polymer and metallic struts in the coronary artery is an attractive alternative strategy to DES-based PCI, as foreign materials could be a source of neointimal hyperplasia, neoatherosclerosis, and thrombosis resulting in stent-related adverse events.^15^^,^^16^ However, concerns for acute vessel closure or restenosis due to recoil have hampered the widespread use of DCB, especially for de novo CAD.^17^ European practice guidelines give a Class I recommendation for the use of DCB in the treatment of ISR but acknowledge that there are no convincing data to support DCB-based PCI for de novo CAD.^18^ In fact, evidence regarding efficacy of DCB-based PCI for de novo CAD has mostly been confined to small-vessel disease, in which use of DES can be challenging.^19^, ^20^, ^21^

Because PCI of complex coronary lesions tends to require more stents as well as longer stents, which can be associated with increased risk of stent undersizing, late malapposition, ISR, and stent thrombosis,^22^^,^^23^ DCB-based PCI could be beneficial for reducing stent burden and stent-related adverse events. However, previous data are limited to several observational studies conducted in specific subsets of complex coronary lesions, and their results are conflicting.^7^^,^^8^^,^^24^^,^^25^ Shin et al^8^ showed that the DCB group had a lower rate of 2-year major adverse cardiovascular events than the DES group (3.9% vs 11.0%) in multivessel disease. Similarly, Gitto et al^25^ reported that DCB-based PCI had a lower incidence of target lesion failure than DES in do novo lesions on the left anterior descending artery. In contrast, 3-year event rates were similar between the DCB and DES groups with diffuse coronary lesions (TLR: 7.3% vs 8.3%, respectively)^7^ and CTO lesions (major adverse cardiovascular events: 12.0% vs 11.8%).^24^ In the current study, DCB-based PCI showed similar rates of 2-year TVF compared with DES-only PCI in various complex coronary artery lesions, including bifurcation lesion, CTO, unprotected LM disease, long lesion, multivessel CAD, lesion requiring ≥3 devices, or severely calcified lesion.

It should be noted that there were some differences in procedural characteristics. First, DCB-based PCI resulted in higher post-PCI diameter stenosis than DES-only PCI. This might be due to the use of relatively smaller devices in the DCB-based group than in the DES-only group or limited radial force and postprocedural recoil of target lesions after DCB-based PCI. Because there was no limitation in reference vessel size in the current study, this might also be due to the operator’s attempt to minimize balloon-induced dissection using smaller size DCB than DES. Nevertheless, the TLR rate after DCB-based PCI was similar to that after DES-only PCI. Considering that previous observational studies and randomized controlled trials reported less angiographic late luminal loss, and even late luminal enlargement, after DCB-based PCI compared with DES-only PCI, relatively suboptimal expansion of the target lesion immediately after DCB-based PCI might have little effect on the future risk of TLR.^26^, ^27^, ^28^ Second, there was no acute vessel closure during the index procedure nor target lesion–related thrombosis during the follow-up period in the DCB-based PCI group. Considering that the risk of definite or probable stent thrombosis was higher after complex PCI than noncomplex PCI,^2^ DCB-based PCI might be a reasonable option in patients with high bleeding risk and complex coronary lesions.^29^ Third, DCB-based PCI was used less frequently for heavily calcified lesions and unprotected LM disease. Considering the higher possibility of suboptimal luminal gain and need for prolonged balloon inflation for delivery of antiproliferative drugs during DCB-based PCI, these subsets of lesions might not be ideal targets for DCB-based PCI.^17^ Conversely, there was a notable propensity of DCB use toward specific types of lesions such as CTO, multivessel CAD, and ostial lesions. Such lesions would be suitable for treatment with DCB because of the presence of collaterals and the benefit of DCB reducing the number of total stents, which is likely to be relatively high in CTO and multivessel disease. Ostial lesions could be treated both by DCB or DES, but DCB might aid in treating the region with size discrepancy between the proximal and distal segment.

Even taking these procedural differences into account, comparable prognosis of DCB-based PCI with DES-only PCI was consistent across various subsets of complex coronary lesions without significant interaction, and DCB-based PCI was not an independent predictor for 2-year TVF in multivariable analysis. In addition, sensitivity analyses of the current study support the comparable efficacy between DCB-based PCI and DES-only PCI. The composition of treated lesion types in each group was significantly different, reflecting daily practice with DCB, and multivariable adjustments for LM disease, CTO, and multivessel PCI did not change the result. The current results imply that DCB-based PCI could be a reasonable treatment strategy to reduce stent burden and stent-related adverse events without concern for increased risk of TVF.^30^ Further study is warranted to confirm these findings and to identify lesion types that would maximize the clinical efficacy of DCB. Ongoing trials such as DCB-HBR (Drug-Coated Balloon in Patients With High Bleeding Risk; NCT05221931) and REVERSE (Drug-Coated Balloon vs. Drug-Eluting Stent for Clinical Outcomes in Patients With Large Coronary Artery Disease; NCT05846893) will provide more evidence of DCB-based PCI for the treatment of de novo CAD.

### Study Limitations

First, because this was a nonrandomized observational study, inherent limitation of selection bias should be considered. Although we used multiple adjustment methods, there were several differences, especially in type of target lesion characteristics. Second, the current results cannot be applied to unprotected LM disease and severely calcified lesions. Third, the current study evaluated 2 years of follow-up data, and longer-term efficacy data of DCB-based PCI are thus not available. Fourth, only paclitaxel-coated DCBs were used in this study, and the results therefore might not be applicable for sirolimus-coated DCBs, as a class effect for DCB cannot be confidently assumed.^18^ Fifth, because the 2 study groups were selected from the various registries of different enrollment periods, variations in practice and other procedure-related factors could have acted as a bias. However, DES of the same generation were used during the total enrollment period, and multiple adjustments have been performed to compensate other discrepancies, such as exclusive inclusion of complex coronary lesion and propensity score matching. Sixth, DCB-based PCIs were heterogeneous, including DCB-only PCI, DCB and DES hybrid PCI, and bail-out DES implantation due to suboptimal DCB angioplasty. Seventh, the data related to bleeding, such as duration of post-PCI antiplatelet agents used and occurrence of severe bleeding complications, were not recorded in the registry, making analysis about the effect of DCB on bleeding impossible in this study. Eighth, some procedural data were not available, such as type of DCB and DES used, type of dissection observed in PCI, and the degree of residual stenosis leading to bailout stenting.

## Conclusions

DCB-based PCI showed comparable risks of TVF vs DES-only PCI in patients with complex coronary artery lesions. This study suggests that DCB might be considered as a suitable alternative device to DES in patients undergoing complex PCI. Further study is needed to validate the current results.

Perspectives **COMPETENCY IN PRACTICE-BASED LEARNING:** Use of DCBs has been considered as an alternative strategy that could reduce the extent and number of DES used during complex PCI, but there are limited clinical data on DCB-based PCI compared with DES-only PCI in patients with various complex coronary artery lesions. The current study showed that DCB-based PCI showed comparable risk of TVF at 2 years with DES-only PCI in patients with de novo complex coronary artery lesions. DCB might be considered as a suitable alternative device to DES in patients undergoing complex PCI.

**TRANSLATIONAL OUTLOOK:** Further randomized controlled trials are needed to validate the efficacy of DCB-based PCI in patients with complex coronary artery lesions and to identify lesion types that would maximize the clinical efficacy of DCB.

## Funding Support and Author Disclosures

Dr J.M. Lee has received an institutional research grant from Abbott Vascular, Boston Scientific, Philips Volcano, Terumo Corporation, Zoll Medical, and Donga-ST. Dr Hahn has received an institutional research grant from the National Evidence-based Healthcare Collaborating Agency, Ministry of Health & Welfare of the Republic of Korea, Abbott Vascular, Biosensors, Boston Scientific, Daiichi-Sankyo, Donga-ST, Hanmi Pharmaceutical, and Medtronic Inc. Dr Gwon has received an institutional research grant from Boston Scientific, Genoss, and Medtronic Inc. All other authors have reported that they have no relationships relevant to the contents of this paper to disclose.
