# Supplementary material for: Drug-Coated Balloon Angioplasty in Patients Undergoing Complex Percutaneous Coronary Intervention
Source: JACC Asia. 2024 Jun 18;4(7):519–31. doi: 10.1016/j.jacasi.2024.04.007 (PMC11291392; doi:10.1016/j.jacasi.2024.04.007)
Supplement: Supplemental Figure 1 and Supplemental Tables 1-5 [file mmc1.docx]

**Supplemental Figure 1. Comparison of 2-Year Risk of Target Vessel Failure Between DCB-based and DES-only PCI According to Clinical Characteristics in Matched Population**

**
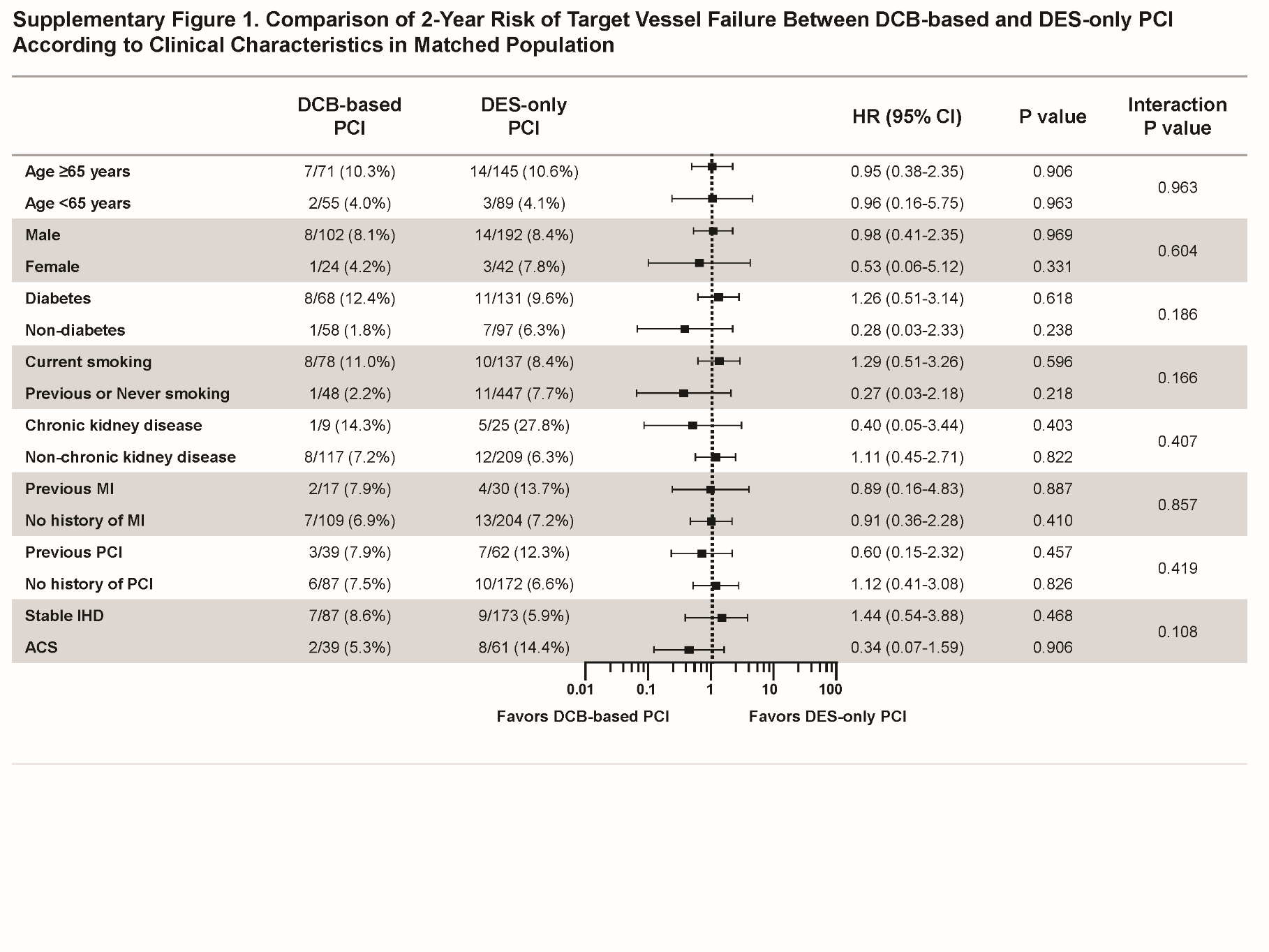
**

Cumulative incidence and HR with 95% CI of TVF at 2 years are presented between DCB-based and DES-only PCI according to clinical characteristics. The interaction P value represents the likelihood of interaction between the variable and the relative treatment effect. The risk of TVF at 2 years was comparable between the two groups across various clinical characteristics without significant interactions. The hazard ratios were calculated with the DES-only group as a reference.

Abbreviations: ACS, acute coronary syndrome; CI, confidence interval; DCB, drug-coated balloon; DES, drug-eluting stent; HR, hazard ratio; IHD, ischemic heart disease; MI, myocardial infarction; PCI, percutaneous coronary intervention.

**Supplemental Table 1. Balance Measurements of Baseline Characteristics in Original Population and Matched Population**

|  | **Original population** | | | **Matched population** | | |
| --- | --- | --- | --- | --- | --- | --- |
|  | **DCB-based PCI** | **DES-only PCI** | **ASMD** | **DCB-based PCI** | **DES-only PCI** | **ASMD** |
| **Patient Characteristics** | 138 (8.5%) | 1478 (91.5%) |  | 126 (35.0%) | 234 (65.0%) |  |
| ***Demographics*** |  |  |  |  |  |  |
| Age (years) | 66.8±10.9 | 63.7±11.2 | 0.257 | 66.2±10.8 | 66.6±10.8 | 0.040 |
| Male | 112 (81.2%) | 1155 (78.1%) | 0.075 | 102 (81.0%) | 192 (82.1%) | 0.028 |
| Body mass index (kg/m^2^) | 24.5±3.1 | 24.6±3.1 | 0.019 | 24.5±3.1 | 24.5±3.0 | 0.013 |
| Baseline LVEF (%) | 58.7±12.2 | 59.6±9.8 | 0.086 | 58.9±12.1 | 59.3±10.8 | 0.034 |
| ***Cardiovascular risk factors*** |  |  |  |  |  |  |
| Hypertension | 96 (69.6%) | 903 (61.1%) | 0.178 | 86 (68.3%) | 168 (71.8%) | 0.077 |
| Diabetes mellitus | 74 (53.6%) | 811 (54.9%) | 0.025 | 68 (54.0%) | 131 (56.0%) | 0.040 |
| Hyperlipidemia | 96 (69.6%) | 492 (33.3%) | 0.777 | 84 (66.7%) | 148 (63.2%) | 0.071 |
| Current smoker | 87 (63.0%) | 736 (49.8%) | 0.269 | 78 (61.9%) | 137 (58.5%) | 0.068 |
| Chronic kidney disease | 11 (8.0%) | 97 (6.6%) | 0.054 | 9 (7.1%) | 25 (10.7%) | 0.124 |
| Peripheral artery disease | 5 (3.6%) | 48 (3.2%) | 0.021 | 5 (4.0%) | 8 (3.4%) | 0.029 |
| Previous myocardial infarction | 25 (18.1%) | 111 (7.5%) | 0.321 | 17 (13.5%) | 30 (12.8%) | 0.020 |
| Previous percutaneous coronary intervention | 51 (37.0%) | 181 (12.2%) | 0.597 | 39 (31.0%) | 62 (26.5%) | 0.098 |
| Previous CABG | 2 (1.4%) | 45 (3.0%) | 0.108 | 2 (1.6%) | 5 (2.1%) | 0.041 |
| Family history of coronary artery disease | 20 (14.5%) | 164 (11.1%) | 0.102 | 18 (14.3%) | 31 (13.2%) | 0.030 |
| ***Clinical presentation*** |  |  | 0.137 |  |  | 0.108 |
| Stable ischemic heart disease | 95 (68.8%) | 921 (62.3%) |  | 87 (69.0%) | 173 (73.9%) |  |
| Acute coronary syndrome | 43 (31.2%) | 557 (37.7%) |  | 39 (31.0%) | 61 (26.1%) |  |
| ***Discharge medication*** |  |  |  |  |  |  |
| Aspirin | 135 (97.8%) | 1360 (92.0%) | 0.267 | 124 (98.4%) | 213 (91.0%) | 0.334 |
| P2Y_12_ Inhibitor | 135 (97.8%) | 1379 (93.3%) | 0.221 | 123 (97.6%) | 218 (93.2%) | 0.213 |
| Beta-blocker | 48 (34.8%) | 649 (43.9%) | 0.187 | 44 (34.9%) | 96 (41.0%) | 0.126 |
| RAAS blockade | 61 (44.2%) | 685 (46.3%) | 0.043 | 55 (43.7%) | 113 (48.3%) | 0.093 |
| Statin | 126 (91.3%) | 1361 (92.1%) | 0.028 | 116 (92.1%) | 216 (92.3%) | 0.009 |
| ***Angiographic evaluation*** |  |  |  |  |  |  |
| Extent of disease |  |  | 0.234 |  |  | 0.028 |
| 1 vessel disease | 23 (16.7%) | 374 (25.3%) |  | 23 (18.3%) | 43 (18.4%) |  |
| 2 vessel disease | 62 (44.9%) | 660 (44.7%) |  | 57 (45.2%) | 110 (47.0%) |  |
| 3 vessel disease | 53 (38.4%) | 444 (30.0%) |  | 46 (36.5%) | 81 (34.6%) |  |
| Multivessel disease | 115 (83.3%) | 1104 (74.7%) | 0.213 | 103 (81.7%) | 191 (81.6%) | 0.003 |
| LM disease | 19 (13.8%) | 198 (13.4%) | 0.011 | 17 (13.5%) | 33 (14.1%) | 0.018 |

Data are expressed as number (%) or mean ± standard deviation.

Abbreviations: ASMD, absolute standardized mean difference; CABG, coronary artery bypass graft; DCB, drug-coated balloon; DES, drug-eluting stent; LM, left main; LVEF, left ventricular ejection fraction; PCI, percutaneous coronary intervention; RAAS, renin-angiotensin-aldosterone system.

**Supplemental Table 2. Comparison of Target Vessel and Target Lesion Characteristics According to Treatment Strategy in Original Population**

|  | **Total**  **(n = 1616)** | **DCB-based PCI**  **(n = 138)** | **DES-only PCI**  **(n = 1478)** | **P value** |
| --- | --- | --- | --- | --- |
| Target vessel location |  |  |  | <0.001 |
| LAD | 724 (44.8%) | 41 (29.7%) | 683 (46.2%) |  |
| LCX | 273 (16.9%) | 66 (48.6%) | 207 (14.0%) |  |
| RCA | 428 (26.5%) | 28 (20.3%) | 400 (27.1%) |  |
| LM | 187 (11.6%) | 3 (2.2%) | 184 (12.4%) |  |
| Graft vessel | 4 (0.2%) | 0 (0.0%) | 4 (0.3%) |  |
| Number of lesion(s) (per patient) | 2.9±1.6 | 3.5±1.8 | 2.8±1.5 | <0.001 |
| Number of target lesion(s) (per patient) | 1.8±0.9 | 1.4±0.6 | 1.9±0.9 | <0.001 |
| Total number of target lesions | 2915 | 198 | 2717 |  |
| Target lesion characteristics (per lesion) |  |  |  |  |
| ACC/AHA type B2/C | 2049 (71.3%) | 146 (73.7%) | 1903 (71.2%) | 0.490 |
| Culprit lesion of ACS | 96 (3.3%) | 2 (1.0%) | 94 (3.5%) | 0.096 |
| Bifurcation lesion | 703 (24.2%) | 39 (19.7%) | 664 (24.5%) | 0.149 |
| Non-LM true bifurcation lesion | 305 (10.5%) | 16 (8.1%) | 289 (10.7%) | 0.304 |
| Diffuse lesion (lesion length≥20mm) | 1233 (43.4%) | 100 (50.5%) | 1133 (42.9%) | 0.044 |
| Thrombotic lesion | 115 (4.0%) | 3 (1.5%) | 112 (4.1%) | 0.102 |
| Chronic total occlusion lesion | 372 (12.8%) | 39 (19.7%) | 333 (12.3%) | 0.004 |
| Ostial lesion | 314 (10.8%) | 33 (16.7%) | 281 (10.4%) | 0.008 |
| Calcification (moderate or severe) | 380 (13.1%) | 17 (8.6%) | 363 (13.4%) | 0.067 |
| Pre-PCI mean diameter stenosis (%) | 86.1±10.1 | 87.2±11.1 | 85.9±10.0 | 0.191 |
| Post-PCI mean diameter stenosis (%) | 3.6±9.6 | 6.2±9.3 | 3.3±9.6 | 0.001 |
| Mean lesion length of target vessel (mm) | 22.9±12.6 | 23.8±12.6 | 22.8±12.6 | 0.352 |
| Total lesion length of target vessel (mm) | 38.8±25.7 | 35.4±26.5 | 39.2±25.6 | 0.097 |

Data are expressed as number (%) or mean ± standard deviation.

Abbreviations: ACC, American College of Cardiology; ACS, acute coronary syndrome; AHA, American Heart Association; DCB, drug-coated balloon; DES, drug-eluting stent; LAD, left anterior descending; LCX, left circumflex; LM, left main; PCI, percutaneous coronary intervention; RCA, right coronary artery.

**Supplemental Table 3. Comparison of Procedural Characteristics According to Treatment Strategy in Original Population**

|  | **Total**  **(n = 1616)** | **DCB-based PCI**  **(n = 138)** | **DES-only PCI**  **(n = 1478)** | **P value** |
| --- | --- | --- | --- | --- |
| Total fluoroscopy time (min) | 22.2±15.6 | 26.1±15.6 | 21.8±15.5 | 0.002 |
| Multivessel PCI | 950 (58.8%) | 103 (74.6%) | 847 (57.3%) | <0.001 |
| Use of intravascular imaging | 443 (27.4%) | 73 (52.9%) | 370 (25.0%) | <0.001 |
| PCI of CTO lesion | 342 (21.2%) | 35 (25.4%) | 307 (20.8%) | 0.249 |
| PCI of bifurcation lesion | 662 (41.0%) | 38 (27.5%) | 624 (42.2%) | 0.001 |
| PCI of unprotected LM disease | 236 (14.6%) | 3 (2.2%) | 233 (15.8%) | <0.001 |
| Use of rotational atherectomy | 62 (3.8%) | 1 (0.7%) | 61 (4.1%) | 0.079 |
| Mean number of devices used | 1.9±0.9 | 2.6±1.3 | 1.8±0.9 | <0.001 |
| Adjunctive dilatation | 522 (32.3%) | 23 (16.7%) | 499 (33.8%) | <0.001 |
| Mean diameter of used devices in target lesion (mm) | 2.98±0.42 | 2.63±0.34 | 3.01±0.41 | <0.001 |
| Total length of used devices in target lesion (mm) | 47.3±25.5 | 42.6±28.2 | 47.7±25.2 | 0.024 |
| Post-PCI TIMI grade 3 | 1559 (96.5%) | 136 (98.6%) | 1423 (96.3%) | 0.253 |
| Bailout stenting | 4 (0.2%) | 4 (2.9%) | 0 (0.0%) | <0.001 |
| Peri-procedural complication | 57 (3.5%) | 4 (2.9%) | 53 (3.6 %) | 0.859 |
| No reflow or Slow flow | 19 (1.2%) | 4 (2.9%) | 15 (1.0%) | 0.121 |
| Cardiogenic shock | 2 (0.1%) | 0 (0.0%) | 2 (0.1%) | >0.999 |
| Cardiopulmonary resuscitation | 5 (0.3%) | 0 (0.0%) | 5 (0.3%) | >0.999 |
| Defibrillation | 2 (0.1%) | 0 (0.0%) | 2 (0.1%) | >0.999 |
| Use of IABP | 16 (1.0%) | 0 (0.0%) | 16 (1.1%) | 0.436 |
| Use of PCPS | 16 (1.0%) | 0 (0.0%) | 16 (1.1%) | 0.436 |
| Stroke | 1 (0.1%) | 0 (0.0%) | 1 (0.1%) | >0.999 |
| Procedural success | 1598 (98.9%) | 134 (97.1%) | 1464 (99.1%) | 0.096 |

Data are expressed as number (%) or mean ± standard deviation.

Abbreviations: CTO, chronic total occlusion; DCB, drug-coated balloon; DES, drug-eluting stent; IABP, intra-aortic balloon pump; LM, left main; PCI, percutaneous coronary intervention; PCPS, percutaneous cardiopulmonary support; TIMI, thrombolysis in myocardial infarction.

**Supplemental Table 4. Independent Predictors for Target Vessel Failure in Original Population**

| **Variable** | **Univariable analysis** | | **Multivariable analysis** | |
| --- | --- | --- | --- | --- |
|  | **HR (95% CI)** | **P value** | **HR (95% CI)** | **P value** |
| DCB-based PCI | 1.31 (0.73-2.33) | 0.362 | 1.18 (0.59-2.36) | 0.643 |
| Age | 1.03 (1.02-1.05) | 0.001 | 1.02 (1.01-1.04) | 0.045 |
| Male | 0.87 (0.57-1.35) | 0.549 | 1.04 (0.63-1.72) | 0.870 |
| Hypertension | 1.23 (0.84-1.82) | 1.000 | 0.81 (0.54-1.24) | 0.334 |
| Diabetes mellitus | 2.40 (1.58-3.65) | <0.001 | 2.03 (1.32-3.12) | 0.001 |
| Hyperlipidemia | 0.79 (0.53-1.18) | 0.247 | 0.92 (0.61-1.40) | 0.702 |
| Current smoker | 0.76 (0.52-1.10) | 0.141 | 0.85 (0.56-1.30) | 0.454 |
| Chronic kidney disease | 4.48 (2.85-7.03) | <0.001 | 3.68 (2.28-5.93) | <0.001 |
| Previous myocardial infarction | 1.58 (0.90-2.76) | 0.112 | 1.06 (0.55-2.03) | 0.871 |
| Previous PCI | 1.69 (1.08-2.65) | 0.023 | 1.25 (0.73-2.13) | 0.418 |
| Clinical presentation (ACS) | 1.17 (0.81-1.71) | 0.408 | 1.04 (0.71-1.52) | 0.839 |
| Multivessel disease | 2.18 (1.26-3.75) | 0.005 | 1.75 (0.93-3.29) | 0.082 |
| Left main disease | 1.93 (1.24-3.01) | 0.004 | 1.81 (1.14-2.86) | 0.012 |
| Multivessel PCI | 1.28 (0.87-1.88) | 0.217 | 0.88 (0.57-1.37) | 0.576 |
| Angiographic success of target vessel | 0.82 (0.36-1.87) | 0.635 | 1.02 (0.40-2.59) | 0.963 |

Abbreviations: ACS, acute coronary syndrome; CI, confidence interval; DCB, drug-coated balloon; HR, hazard ratio; PCI, percutaneous coronary intervention.

**Supplemental Table 5. Sensitivity Analysis Regarding Clinical Outcomes According to Treatment Strategy in Original Population**

|  | **Unadjusted HR**  **(95% CI)** | **IPW adjusted HR**  **(95% CI)** | **PS adjusted HR**  **(95% CI)** | **PS stratified HR**  **(95% CI)** |
| --- | --- | --- | --- | --- |
| Patient Number | 1616 | 1616 | 1616 | 1616 |
| TVF* | 1.31 (0.73-2.33) | 0.56 (0.29-1.09) | 0.94 (0.48-1.81) | 1.00 (0.06-15.99) |
| Cardiac Death | 2.01 (0.94-4.28) | 1.06 (0.46-2.41) | 1.62 (0.68-3.89) | NA |
| Target Vessel Myocardial Infarction | 0.42 (0.06-3.10) | 0.20 (0.03-1.45) | 0.53 (0.07-4.21) | NA |
| Target Vessel Revascularization | 1.02 (0.44-2.38) | 0.35 (0.14-0.89) | 0.67 (0.26-1.74) | 1.00 (0.06-15.99) |

^*^TVF defined as a composite of cardiac death, target vessel myocardial infarction, target vessel revascularization.

Abbreviations: CI, confidence interval; HR, hazard ratio; IPW, inverse probability weighting; NA, not available; PS, propensity score; TVF, target vessel failure.
